# Possible abscopal effect in urothelial carcinoma of the upper urinary tract after treatment with immune checkpoint inhibitors

**DOI:** 10.1002/iju5.12133

**Published:** 2019-12-03

**Authors:** Yudai Ishiyama, Toshio Takagi, Kazuhiko Yoshida, Junpei Iizuka, Yoichi Kakuta, Masayoshi Okumi, Hideki Ishida, Kazunari Tanabe

**Affiliations:** ^1^ Department of Urology Tokyo Women's Medical University Tokyo Japan; ^2^ Department of Organ Transplant Medicine Tokyo Women's Medical University Tokyo Japan

**Keywords:** immunotherapy, lymph nodes, neoplasm metastasis, radiotherapy, urologic neoplasms

## Abstract

**Introduction:**

Regression of non‐irradiated metastatic lesions after radiation therapy is known as the abscopal effect. We report a case of urothelial carcinoma in which the abscopal effect was possibly observed after immune checkpoint inhibitor administration.

**Case presentation:**

A 68‐year‐old woman diagnosed with left renal pelvic cancer underwent total nephroureterectomy and regional lymph node dissection. Eight months later, imaging studies detected local recurrence and paraaortic lymph node metastasis. The tumor progressed despite cisplatin + gemcitabine, pembrolizumab, and gemcitabine + docetaxel therapy. Radiation therapy was administered to a painful back lesion, which resulted in dramatic symptom relief. Computed tomography 2 months after radiation therapy indicated reduced size of the irradiated lesion and some non‐irradiated lymph nodes.

**Conclusion:**

Combined radiation therapy and immune checkpoint inhibitors can provide additional benefits for certain cancers, possibly due to negative immunomodulatory response blockade. Thus, this combined therapy may be a new metastatic urothelial carcinoma treatment strategy.

**Abbreviations & Acronyms**
CT: computed tomography
GC: gemcitabine + cisplatin
GD: gemcitabine + docetaxel
ICI: immune checkpoint inhibitor
LN: lymph node
PD: progressive disease
RT: radiation therapy
UC: urothelial carcinoma

**Keynote message**

The abscopal effect is regression of non‐irradiated metastatic lesions after RT. We describe a case of UC in which the abscopal effect was observed after administration of an ICI. This additional benefit, possibly due to the negative immunomodulatory response blockade, sheds light on the possibility of combination therapy for UCs.

## Introduction

For patients with metastatic UC of the upper urinary tract, systemic chemotherapy is the preferred first‐line treatment, although its effect is limited.^1^ ICIs have emerged as an alternative mode of treatment. We report a unique case, possibly demonstrating the abscopal effect, as shrinkage of non‐irradiated metastatic lesions was observed after administering RT subsequent to chemotherapy and pembrolizumab.

## Case presentation

A 68‐year‐old woman was diagnosed with left renal pelvic cancer by enhanced CT after presenting with gross hematuria. The CT image showed an enhancing mass in the area. Urine cytology revealed class III cells and cystoscopy suggested no apparent tumor in the bladder. She underwent total nephroureterectomy and regional LN dissection with no major perioperative complications. The lesion in the surgical pathology specimen was identified as high‐grade UC (pT2 with lymphovascular invasion) but there was no metastasis to the surrounding LNs (0/7). Eight months after the surgery, follow‐up cystoscopy revealed recurrence within the bladder, which was confirmed as UC upon biopsy analysis. At this time, imaging studies (CT and positron emission tomography/CT) detected local recurrence (surrounding the original kidney) and paraaortic LN enlargement consistent with metastasis.

**Figure 1:**
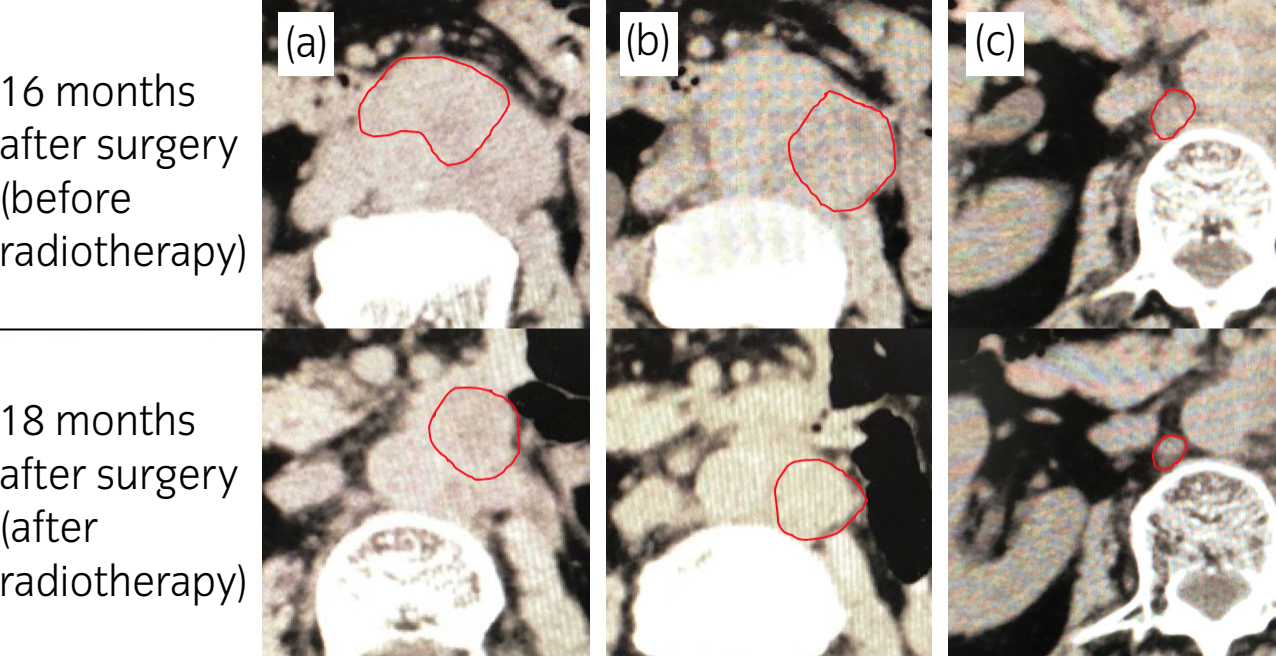
CT showing reduction in metastatic LNs after radiotherapy. (a,b) Paraaortic LNs. (c) Right renal hilum LN. The upper row shows LNs at 16 months after surgery (before radiotherapy); the lower row shows LNs at 18 months after surgery (after radiotherapy). The red line outlines the circumference of target lesions.

**Figure 2:**
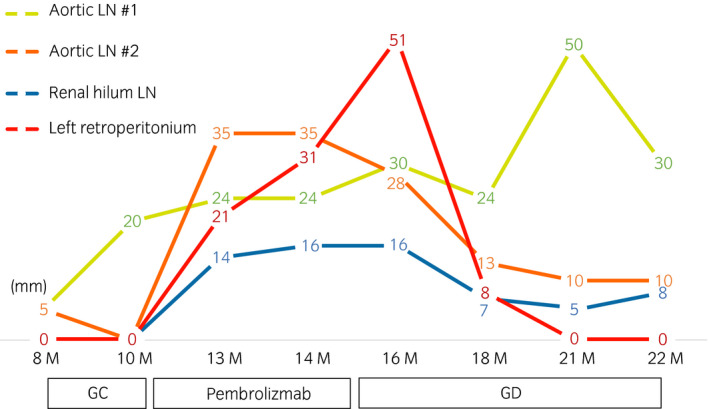
Clinical treatment course.

Chemotherapy (cisplatin + gemcitabine) was administered as the first‐line systemic therapy immediately after the diagnosis was made. Two months later, CT showed an increase in the size of the paraaortic LN consistent with PD, and the treatment was switched to ICI therapy with pembrolizumab. However, subsequent CT scans revealed not only paraaortic LN enlargement but also left subclavian LN and right renal hilum LN enlargement at 3 and 4 months after the initiation of pembrolizumab, respectively, which also satisfied the criteria for PD. Pembrolizumab was discontinued, and after discussing the risks and benefits of each third‐line chemotherapy regimen with the patient, we decided to start GD which resulted in continuous overall tumor growth. At this time, the patient complained of unbearable left back pain, which was considered to be caused by local recurrence invading the surrounding muscles. RT (30 Gy in 10 fractions) was administered to the lesion considered to be the source of the pain; this resulted in dramatic symptom relief. CT 2 months after the initiation of RT showed reduction of the irradiated lesion from 51 to 8 mm as well as reduction of some non‐irradiated LNs, such as two paraaortic LNs (#1: 30–24 mm, #2: 28–13 mm) and the right renal hilar LN (16–7 mm) (Fig. 1). In summary, partial response was achieved even in non‐irradiated lesions after RT. CT performed 21 months post‐operation showed that the irradiated lesion had disappeared (from 8 to 0 mm) and that two of the non‐irradiated lesions remained stable without a decrease in size (paraaortic LN #2: from 13–10 mm and right renal hilum LN: from 7–5 mm). However, one of the aortic LNs (#1) increased in size (from 24 to 50 mm). Therefore, additional RT doses were administered to the aortic LN #1 and tumor shrinkage was observed (from 50 to 30 mm), while the other lesions remained stable (Fig. 2).

## Discussion

The regression of non‐irradiated metastatic lesions with RT is referred to as the abscopal effect. The mechanism is thought to be related to RT‐induced release of tumor antigens, which are taken up by antigen‐presenting cells that activate the major histocompatibility complex pathway along with other signals that eventually activate effector T‐cells, which induce cell death.^2^ The earliest report of the abscopal effect was published in the 1950s and has been reported and investigated in multiple types of cancer.^3^ In recent years, the combination of RT and ICIs has been proven to provide an additional effect in certain types of cancers, possibly from the blockade of the negative immunomodulatory response.^4^, ^5^ Few reports of the abscopal effect in UC exist^6^ and, to the best of our knowledge, this case is the first report of abscopal effect in both renal pelvic cancer and UC in the ICI era. As there is more interest in the role of ICIs for UC management, trials are needed to determine whether they provide survival benefit.

Our patient received two types of chemotherapy before RT administration, and there are several points for discussion. One is that the combination of three different therapies (chemotherapy, ICI, RT) may have caused the abscopal effect. Further trials are required to assess this theory. Another is that the chemotherapy contributed to the release of tumor antigens. However, the fact that shrinkage occurred only after the initiation of RT led us to assume that the RT was responsible for the observed shrinkage of the non‐irradiated lesions.

To the best of our knowledge, there is no concrete definition of the “ICI and RT combination” to date. For other types of malignant tumors, several sequences have been reported in which the abscopal effect was observed, but it remains unclear whether RT before, concurrent with, or after drug administration is the most effective.^7^, ^8^, ^9^ This case involved “sequential therapy with ICI followed by RT,” but the optimal time window for RT and/or ICI chemotherapy is beyond the discussion of this case. Future clinical trials using ICI/RT combination will hopefully further clarify optimal sequential rationale.

## Conclusions

In conclusion, we observed a possible abscopal effect due to RT in a patient with UC after ICI treatment. The combination of RT and ICIs may highlight a new strategy for the treatment of metastatic UC.

## Disclosure of ethical statement

All informed consent was obtained from the patient. This work was conducted in accordance with the Declaration of Helsinki.

## Conflict of interest

The authors declare no conflict of interest.
